# A Huge Osteochondroma With Chest Wall Deformity Arising From the Ventral Scapula

**DOI:** 10.7759/cureus.58275

**Published:** 2024-04-14

**Authors:** Ratnakar Ambade, Chandrashekhar Mahakalkar, Yashika Sharma, Rahul Singh, Kashyap Kanani

**Affiliations:** 1 Orthopaedics, Jawaharlal Nehru Medical College, Datta Meghe Institute of Higher Education and Research, Wardha, IND; 2 General Surgery, Jawaharlal Nehru Medical College, Datta Meghe Institute of Higher Education and Research, Wardha, IND

**Keywords:** scapular osteochondroma, chest wall deformity, osteochondroma, huge osteochondroma, ventral scapular osteochondroma

## Abstract

Osteochondromas (OC), or exostoses, are developmental defects rather than true neoplasms. Misdirected physeal bone growths give rise to OC. It causes cartilage-capped bony extensions to emerge from the lateral outlines of endochondral bones. We discuss a case of OC in a 35-year-old female who presented with severe chest wall deformity and breathlessness due to compromised left lung function. CT scan showed a vast osteochondroma arising from the ventral surface of the scapula, which was palpable in the supra mammary region on the left side. The tumor mass was completely excised from the base of the stalk. Her breathlessness and compromised left lung function returned to normal in the post-op period. However, the chest deformity was corrected over two months. The article provides insights into the presentation in a patient with such a massive tumor due to its location. Surgical excision should be the treatment of choice for huge osteochondromas.

## Introduction

Osteochondroma (OC) is the most common benign bone tumour; its development process remains unclear [1]. Osteochondromas, or exostoses, are developmental defects rather than true neoplasms, and they result from misdirected physeal bone growth, which causes cartilage-capped bony extensions to emerge from the lateral outlines of endochondral bones [2-4]. Metaphyseal regions of long tubular bones, such as in the femur, humerus, and tibia, are the most common sites of OC, while flat bones are less frequently affected [5]. The reported incidence of OC is 33-35% of all benign tumours [5,6]. Although the scapula is a rare site, OC is the most common tumour of the scapula [2,3]. Osteochondromas often cause no discomfort; the location and size of the OC determine the symptoms that arise. Difficulties such as a mass effect usually result in mechanical issues; bone stalk fractures; nerve impingement; compression over blood vessels, nerves, or the spinal cord; bursa development; and malignant cartilage cap transformation [5,6]. We describe a case of a 35-year-old female with severe chest wall deformity and breathlessness due to compromised left lung function. It was caused by a huge OC arising from the inferolateral part of the ventral surface of the scapula.

## Case presentation

**Figure 1:**
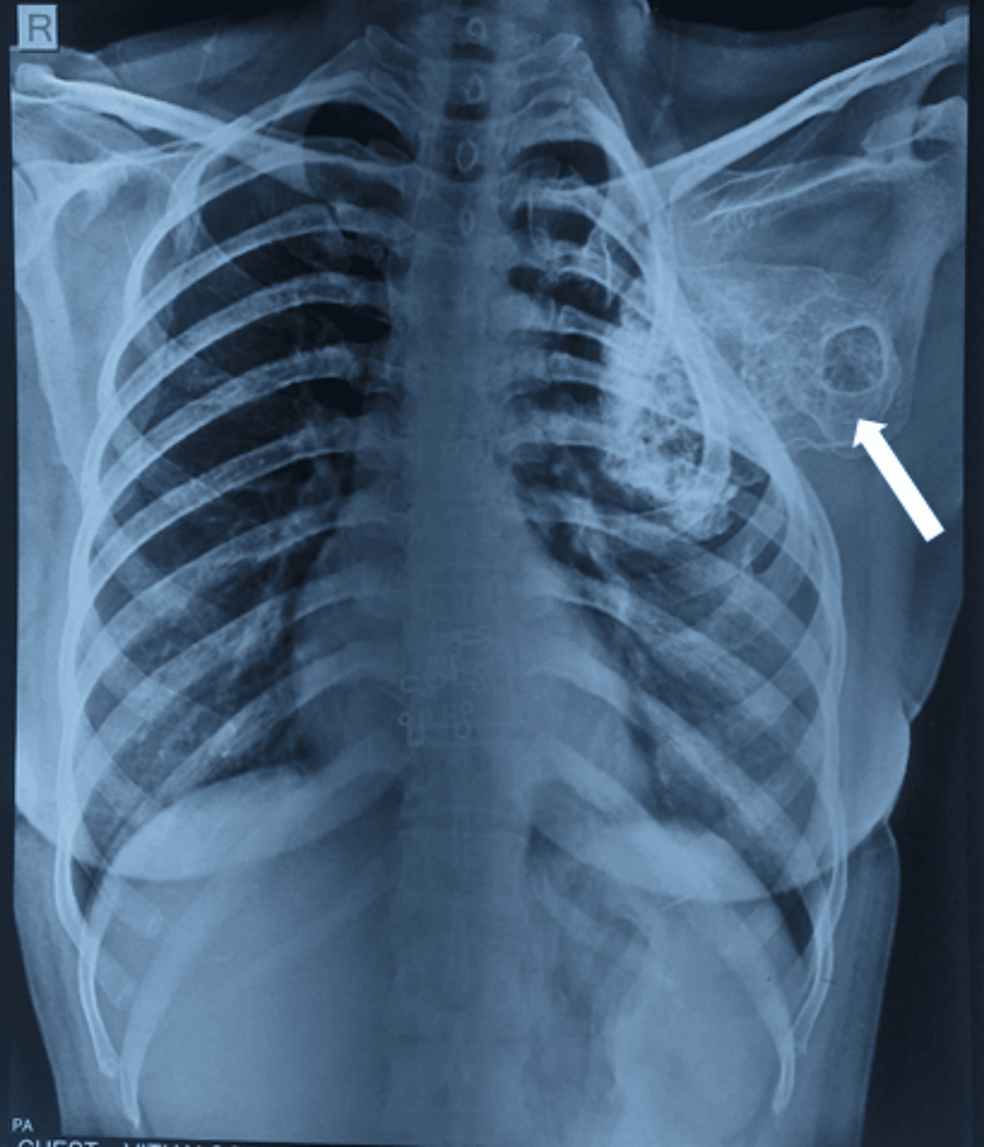
Chest X-ray showing the chest wall deformity and the tumor mass arising from the scapula

The patient was a 35-year-old woman who presented with breathlessness for six months, which had been aggravating for one month, and a hard palpable mass in the left supra mammary region for two years. She also reported restricted shoulder abduction with pain in the axilla and scapular region, more so during movements while doing her daily activities for one year. She gave a history of palpable mass in the left axilla since she was 20 years old. A hard palpable mass in the left supra mammary region of 5 x 4 cm was felt on physical examination. Similarly, the same mass was felt 6 x 4 cm in size in the axilla. This bony hard mass was mobile with scapular movements and was more prominent on abduction. Shoulder movements were possible with discomfort, abduction range of motion was 0-45 degrees terminally restricted and painful, external rotation and flexion were limited, and regional lymph nodes were not palpable. The left shoulder was lower than the contralateral shoulder, and there was no relevant family or medical history. Neurological examination was normal. There was no winging of the scapula. There was no atrophy of the back muscles and contraction of the trapezius. The short rotators and rotator cuff were non-tender and without any defects. Anteroposterior plain radiograph revealed a sclerotic mass arising from the lower inferolateral surface of the left scapula on the ventral side and a chest wall deformity of the left side caused by the bony mass due to compression. The mass abutted the seventh and eighth rib and displaced them at the anterior end, as shown in Figure 1.

**Figure 2:**
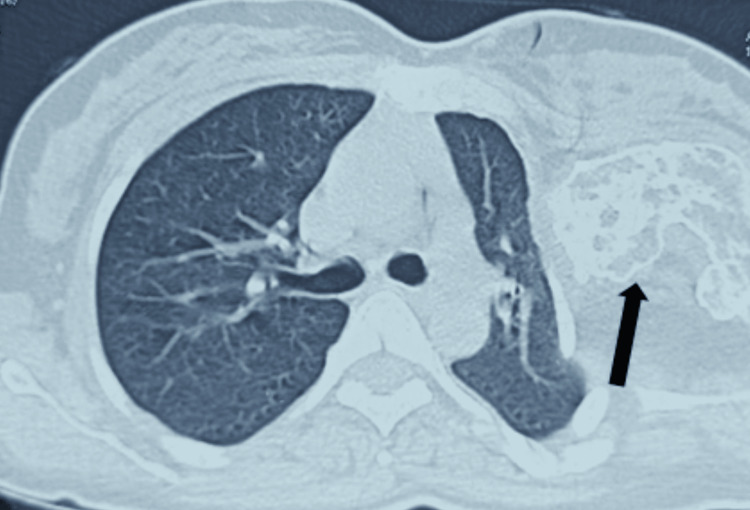
CT scan showing the compromised left lung and the tumor mass compressing the left lung CT: computed tomography

A CT axial view revealed a compromised lung on the left side due to the mass compressing the lung, as shown in Figure 2.

**Figure 3:**
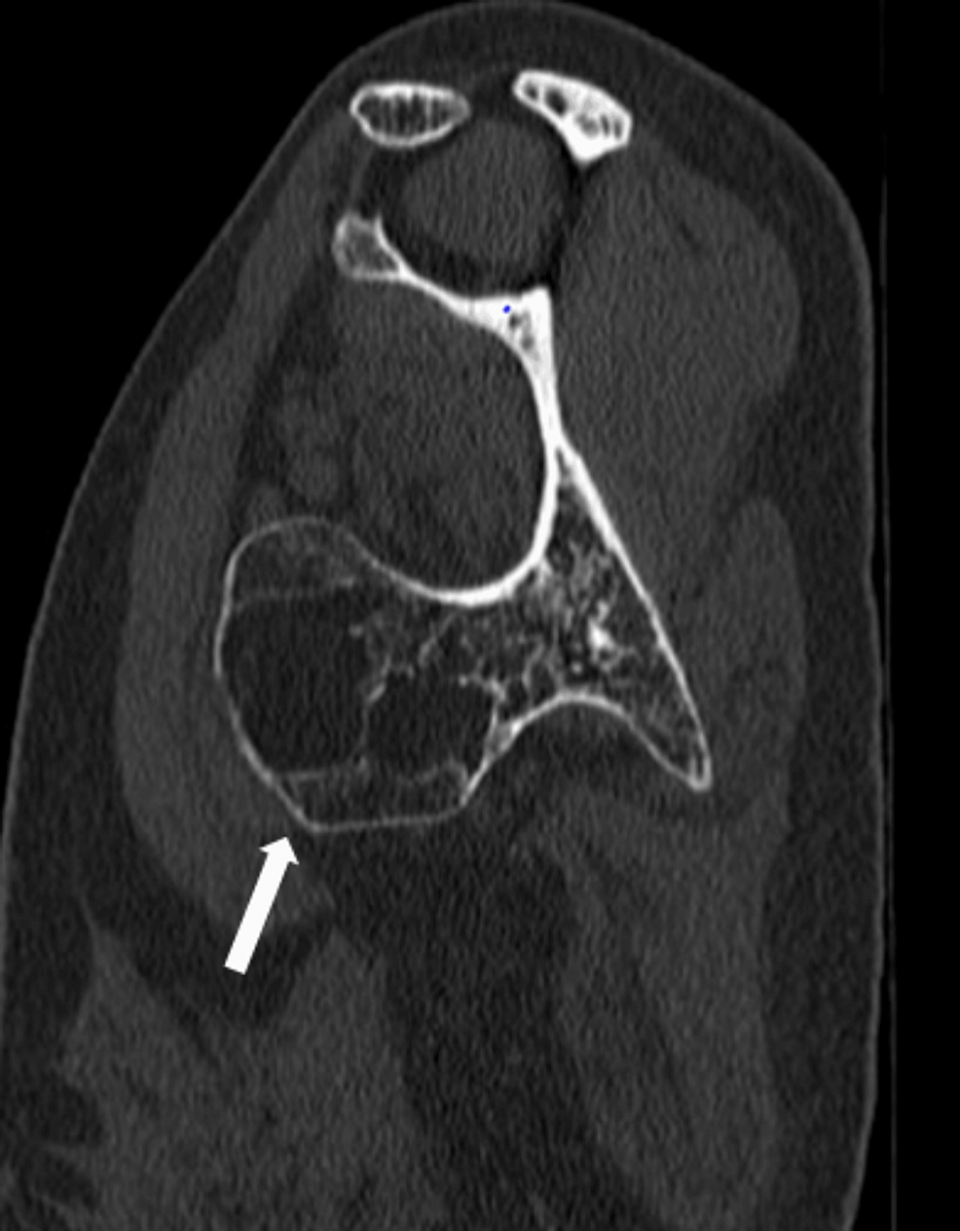
CT scan showing the bony mass with the stalk attached to the scapula CT: computed tomography

**Figure 4:**
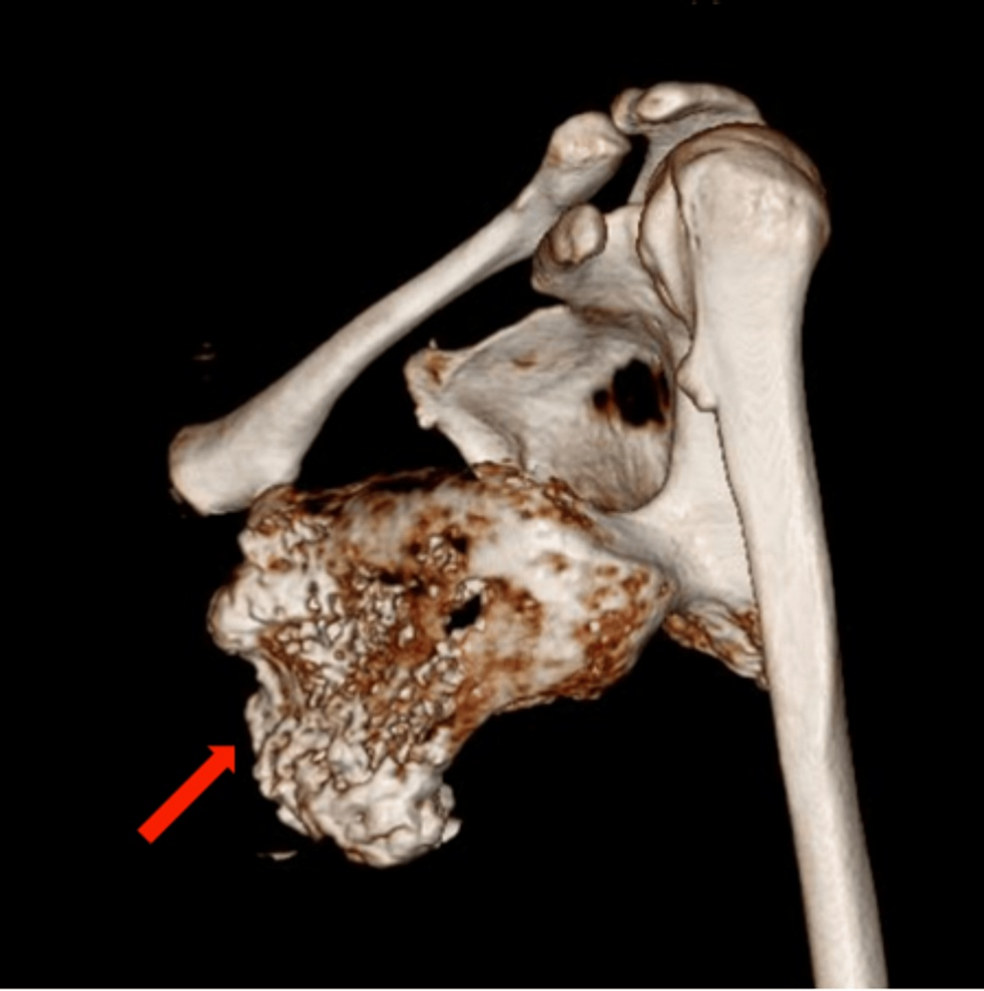
Three-dimensional CT scan showing the bony mass with the stalk attached to the scapula CT: computed tomography

The 3D CT showed a bony protrusion arising from the inferolateral part of the ventral surface of the scapula. The mass, measuring 13 x 8 x 6 cm, was directed anteriorly and superiorly towards the chest wall abutting the seventh and eighth ribs, as shown in Figure 3 and Figure 4.

Excision of the tumor mass

**Figure 5:**
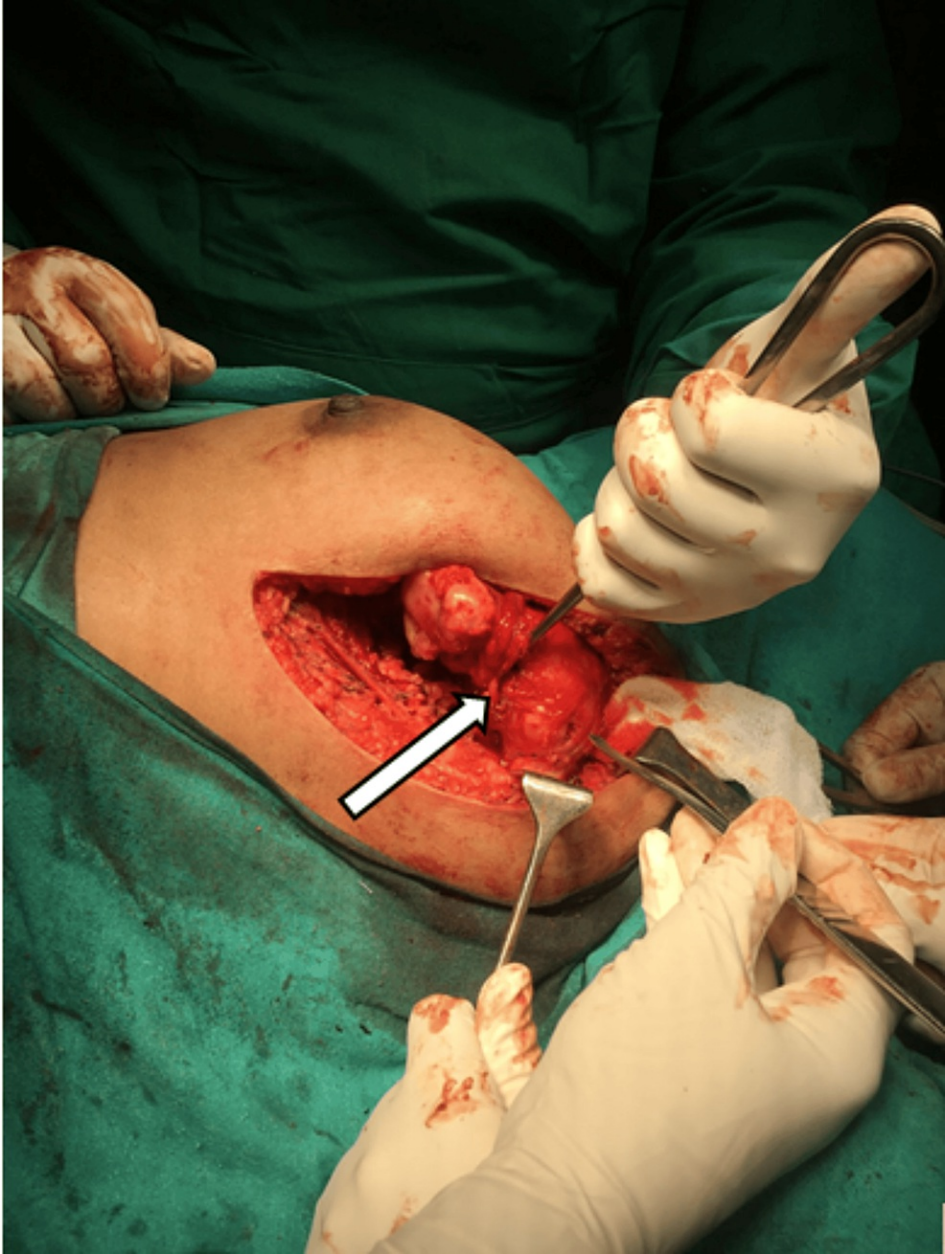
Intraoperative image showing the osteochondroma before excision

The tumor mass was difficult to access as it was arising from the inferolateral part of the ventral surface of the scapula and progressing anteriorly to the supra mammary region. It would have been difficult to access the anterior part and avoid injury to the pleura through the posterior approach alone. Hence, the mass was approached through a lateral mammary incision. The patient was given general anesthesia with single lung ventilation. She was placed supine with a sandbag under the scapula to elevate it. A lateral mammary incision was made, which was extended up to the anterior axillary fold. The incision was deepened, and the lateral border of the pectoralis major was identified. A subpectoral plane was developed. The pectoralis major was elevated to expose the underlying mass. The inferior attachments of the pectoralis major muscle over the seventh, eighth, and ninth ribs were released to attain total exposure. The mass released from its attachments to the lateral and anterior surfaces was identified. The medial and inferior surfaces were attached over the pleura. The mass was exposed entirely to the stalk, as shown in Figure 5.

**Figure 6:**
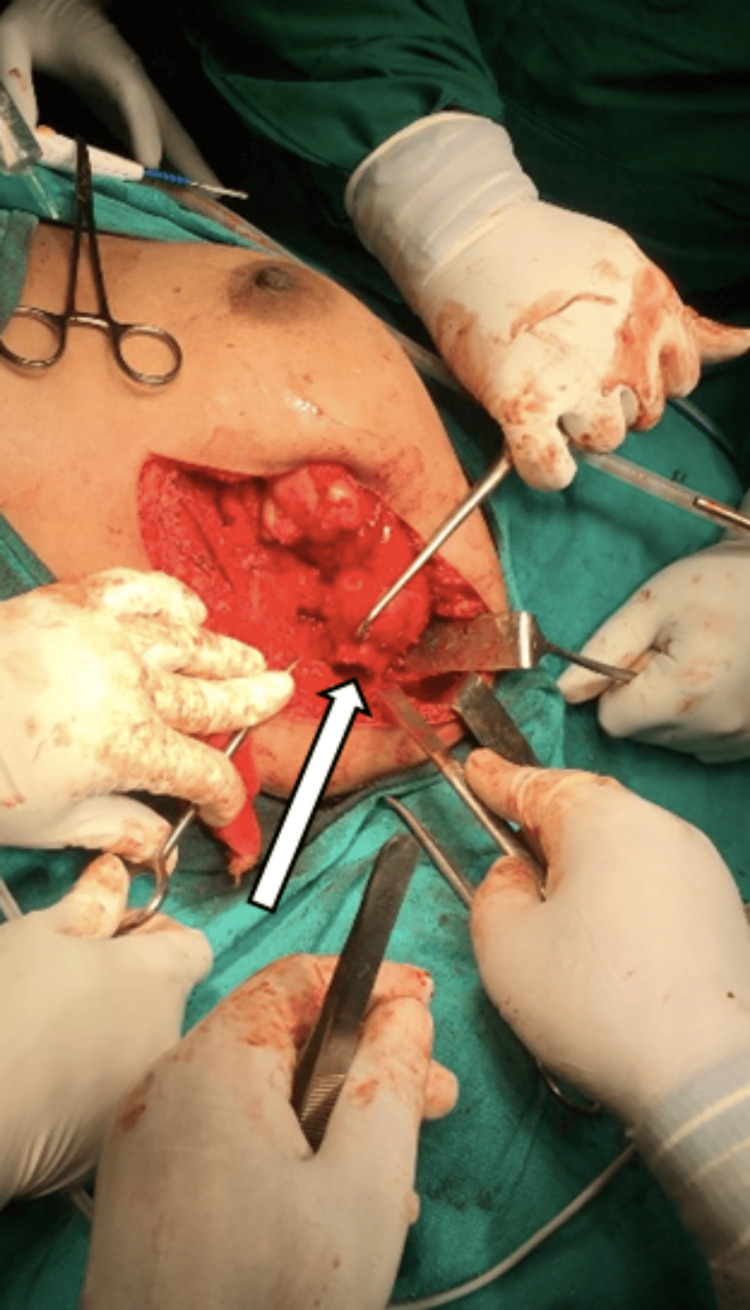
Osteochondroma was excised at the base of the stalk

The tumor stalk was excised at the base with an osteotome and hammer as shown in Figure 6.

**Figure 7:**
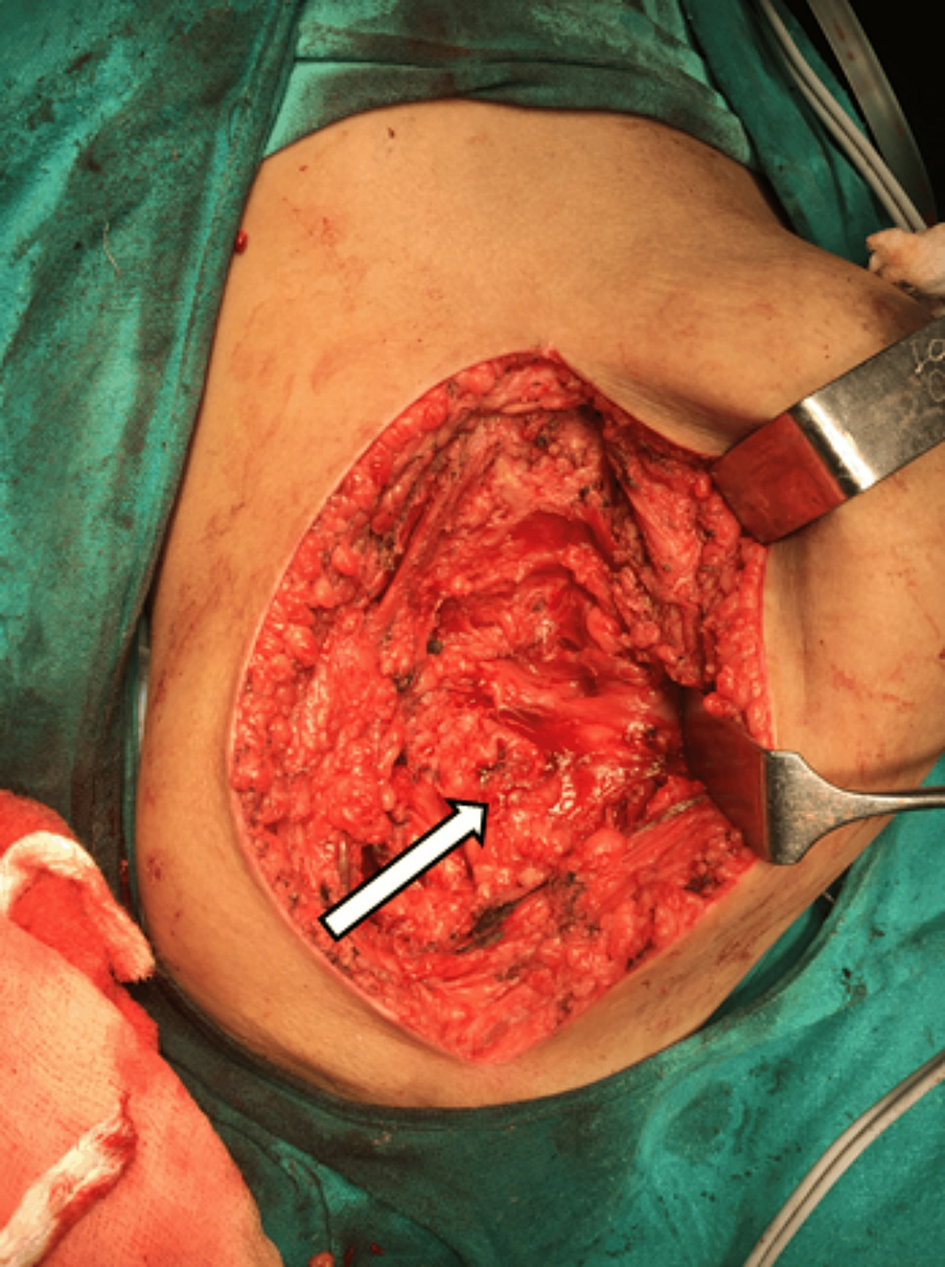
Tumour mass was removed and hemostasis achieved

**Figure 8:**
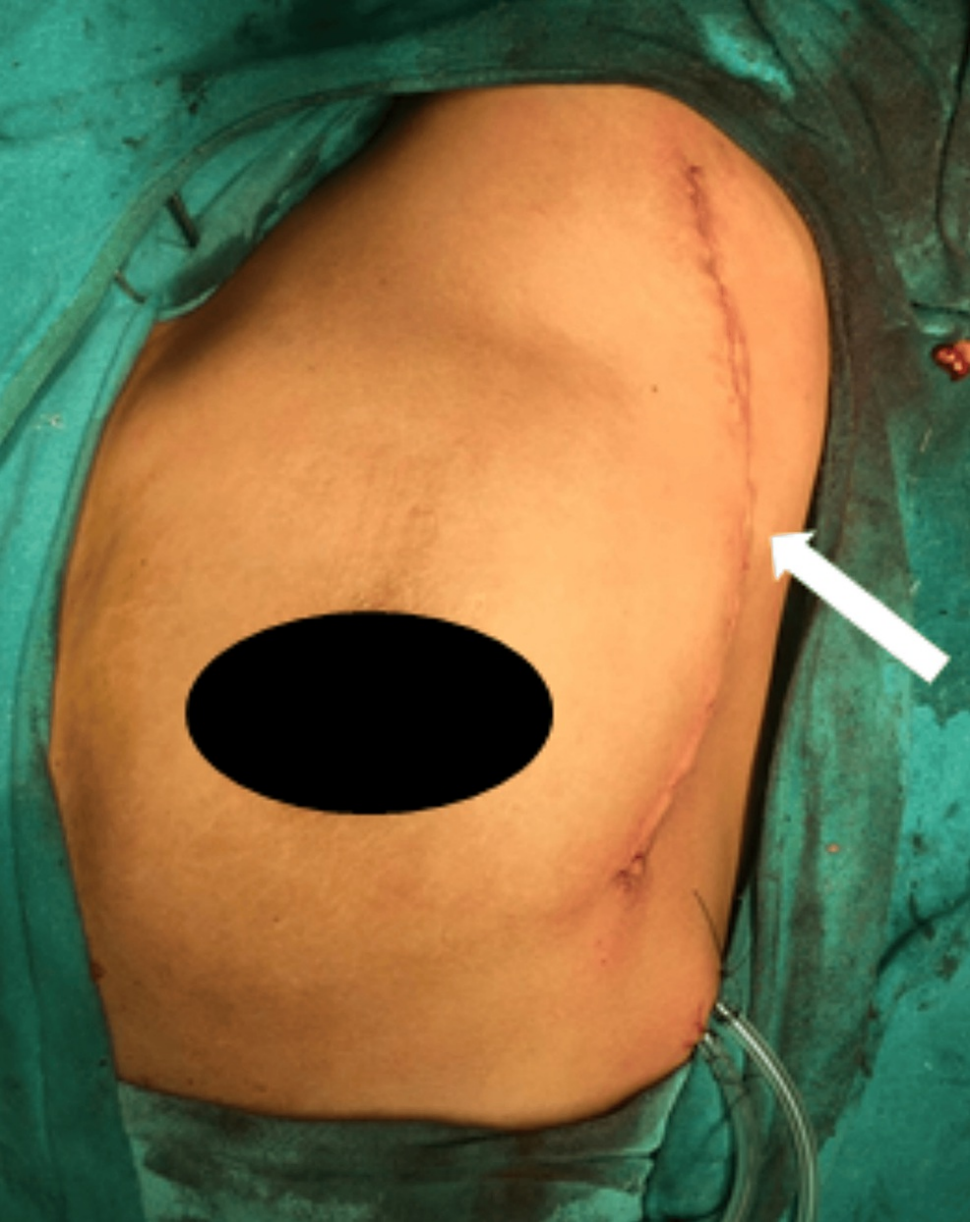
Lateral mammary incision after closure

Holding and lifting the tumor mass with a bone hook, the medial and inferior surfaces were released of attachments, and attention was paid not to injure the pleura. The tumor was completely removed. Hemostasis was achieved. Wound closure was done over a negative suction drain with subcuticular stitches, as shown in Figure 7 and Figure 8.

**Figure 9:**
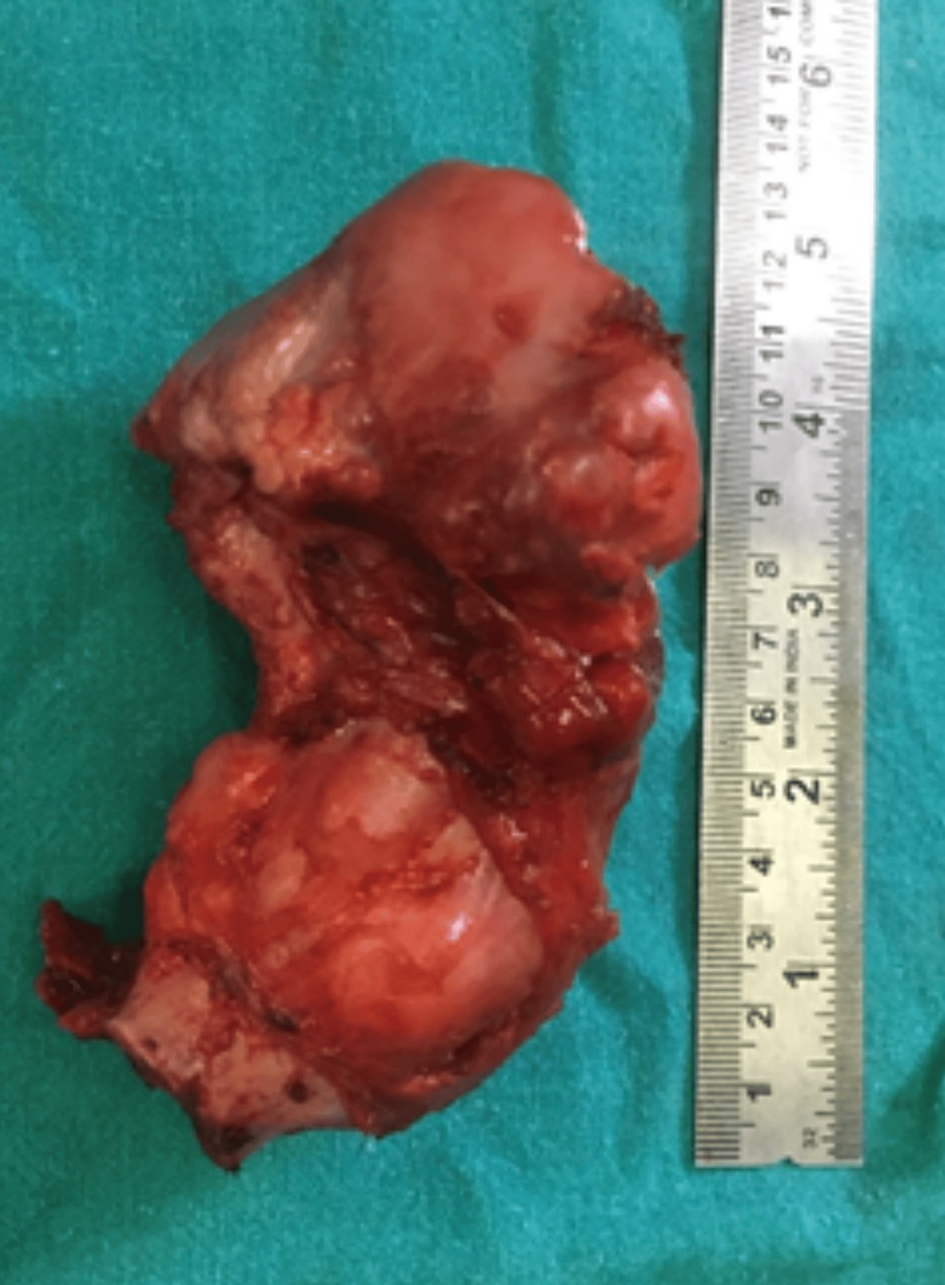
The excised tumor mass

The excised tumour mass measured 13 x 8 x 6 cm in size. Macroscopically, it was a pedunculated, irregular, pearly white nodular hard mass, as shown in Figure 9.

**Figure 10:**
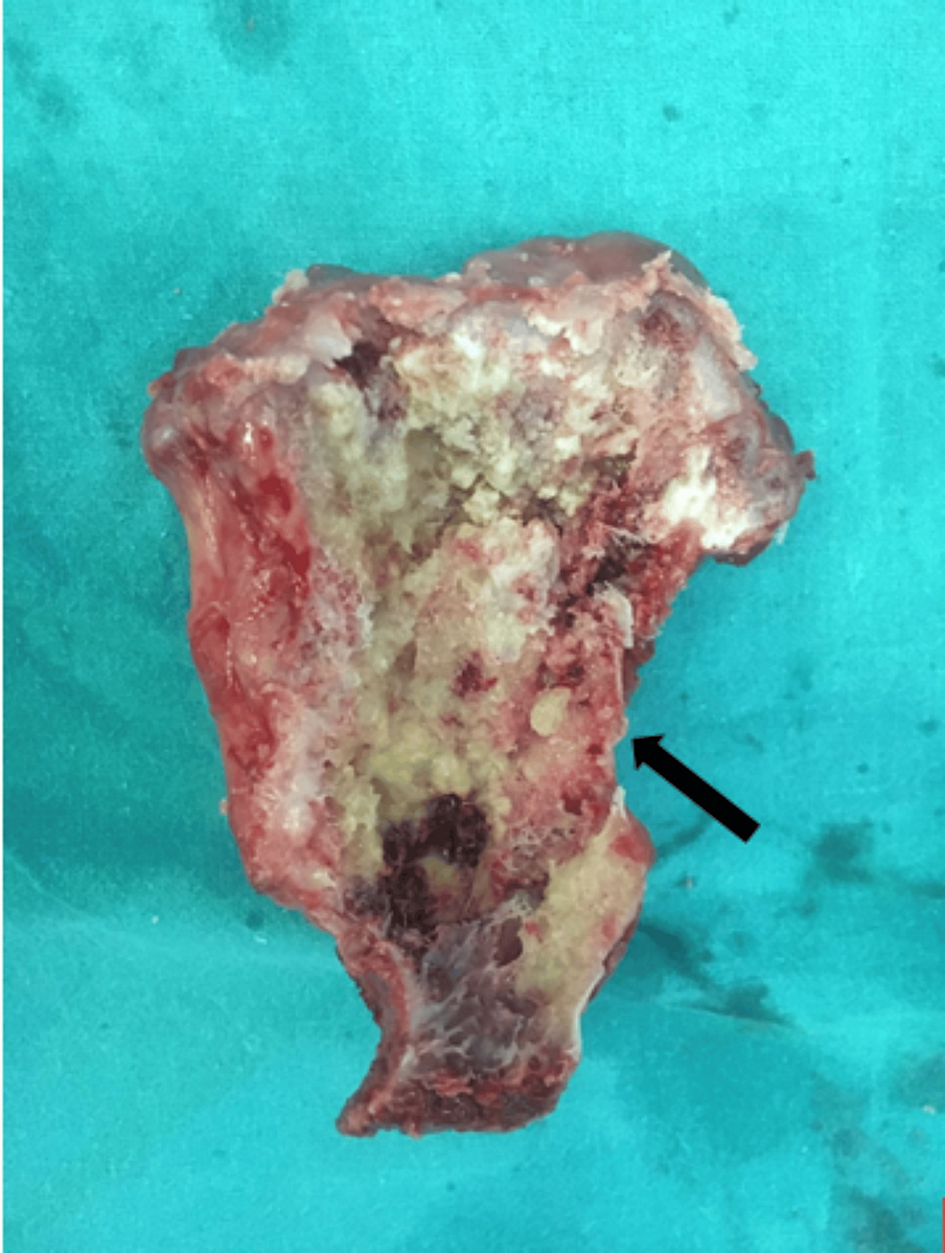
Cut section of the tumor mass showing a cartilaginous cap and fatty bone marrow

The cut surface revealed a spongy bone with a thin <1 cm cartilaginous cap, as shown in Figure 10.

**Figure 11:**
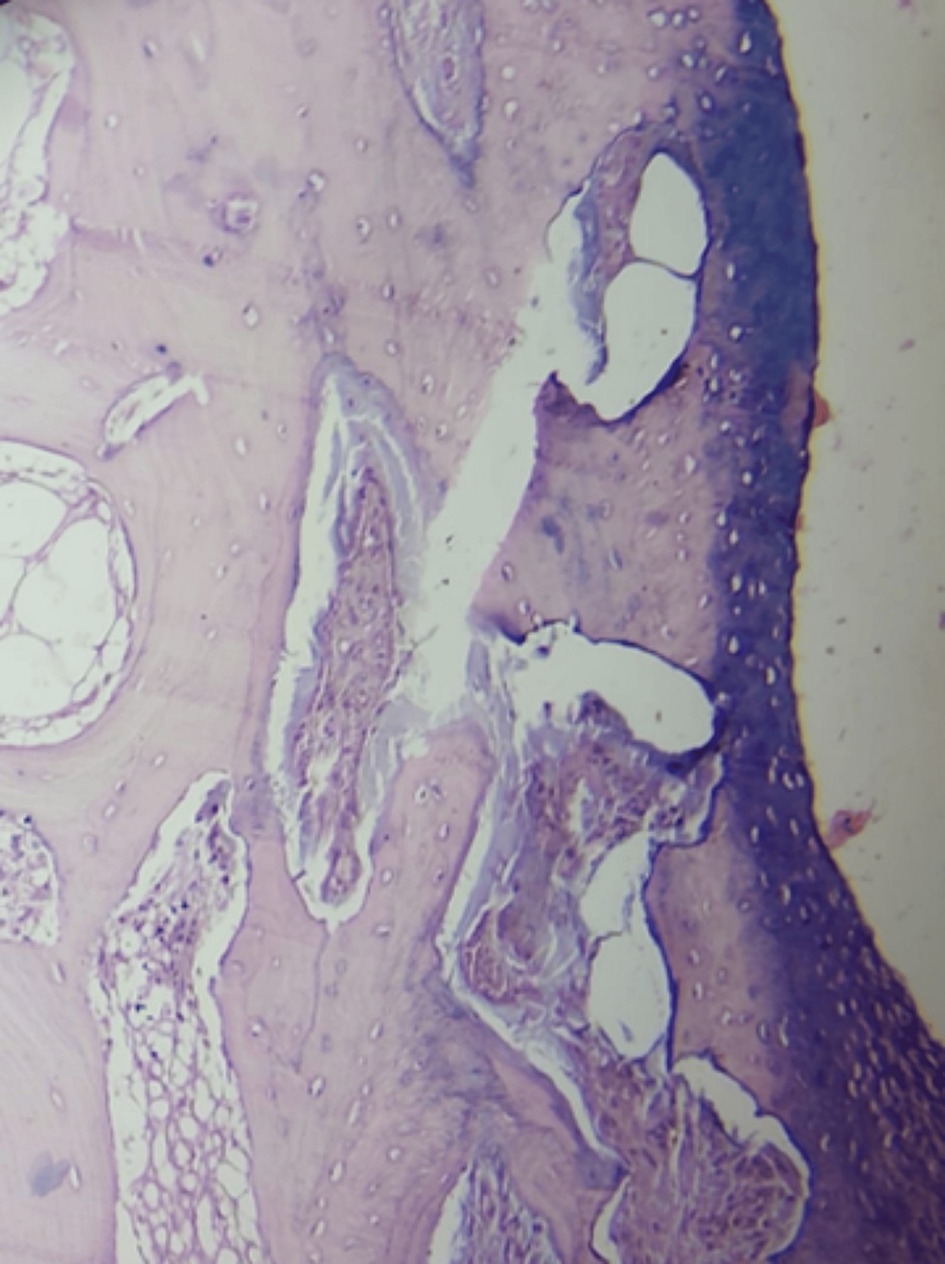
Histopathological examination showing hyaline cartilaginous cap overlying fibrous perichondrium

The histopathological evaluation confirmed the diagnosis of osteochondroma with no evidence of malignant transformation. Microscopic examination revealed a hyaline cartilaginous cap overlying fibrous perichondrium. Bony areas with marrow elements were observed, as shown in Figure 11.

Outcome and follow-up

**Figure 12:**
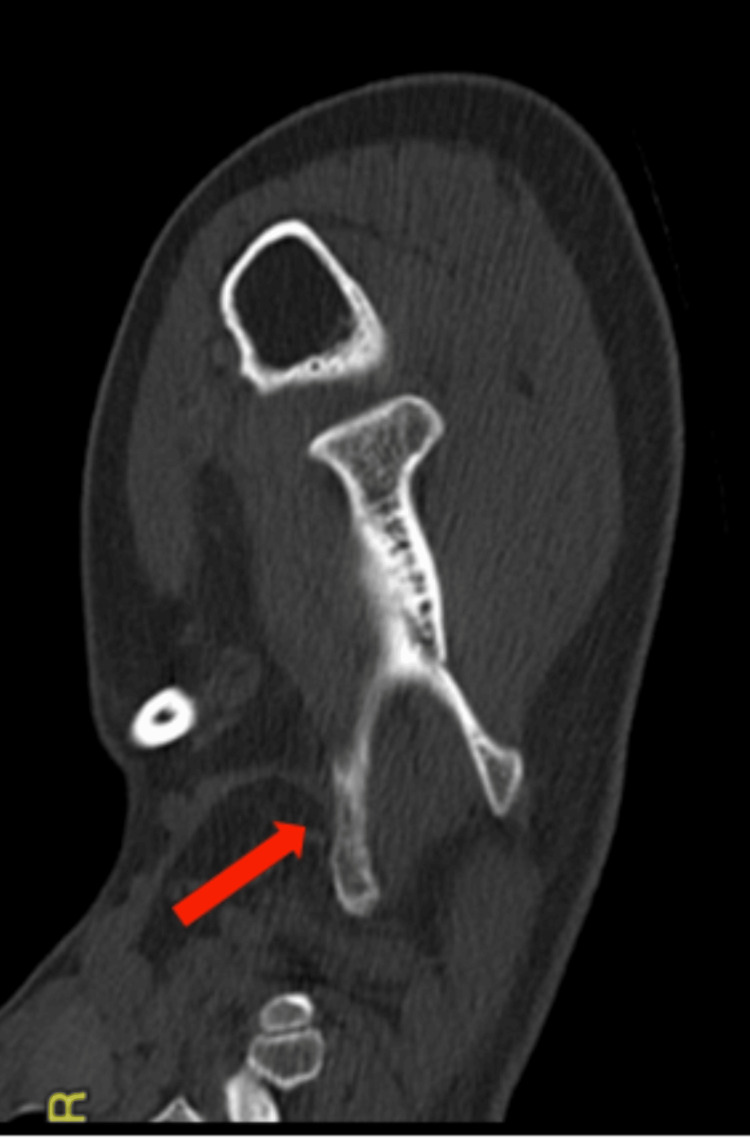
Postoperative CT scan showing the excised tumor mass CT: computed tomography

**Figure 13:**
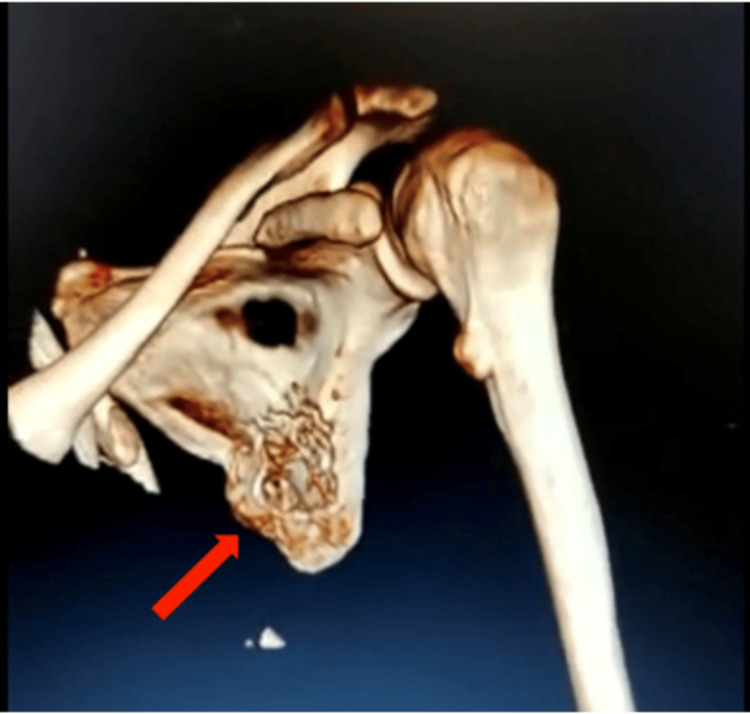
Three-dimensional CT scan showing the excised tumor mass CT: computed tomography

Postoperatively, the patient's respiratory distress was relieved. The arm was immobilized in an arm pouch for three weeks, during which pendulum exercises were started for the shoulder. Strengthening exercises for the scapular stabilizers and respiratory exercises were started immediately from the second postoperative day onwards. The patient was maintaining 98-99% oxygen saturation without any assistance. She was comfortable with respiration and shoulder movements in the postoperative period. However, the chest X-ray in the postoperative period still showed the deformity of the chest wall, but there was no compressive effect, and the patient's respiration was comfortable. Postoperative radiographs and CT revealed complete excision of osteochondroma, as shown in Figure 12, as well as the 3D CT scan (Figure 13).

**Figure 14:**
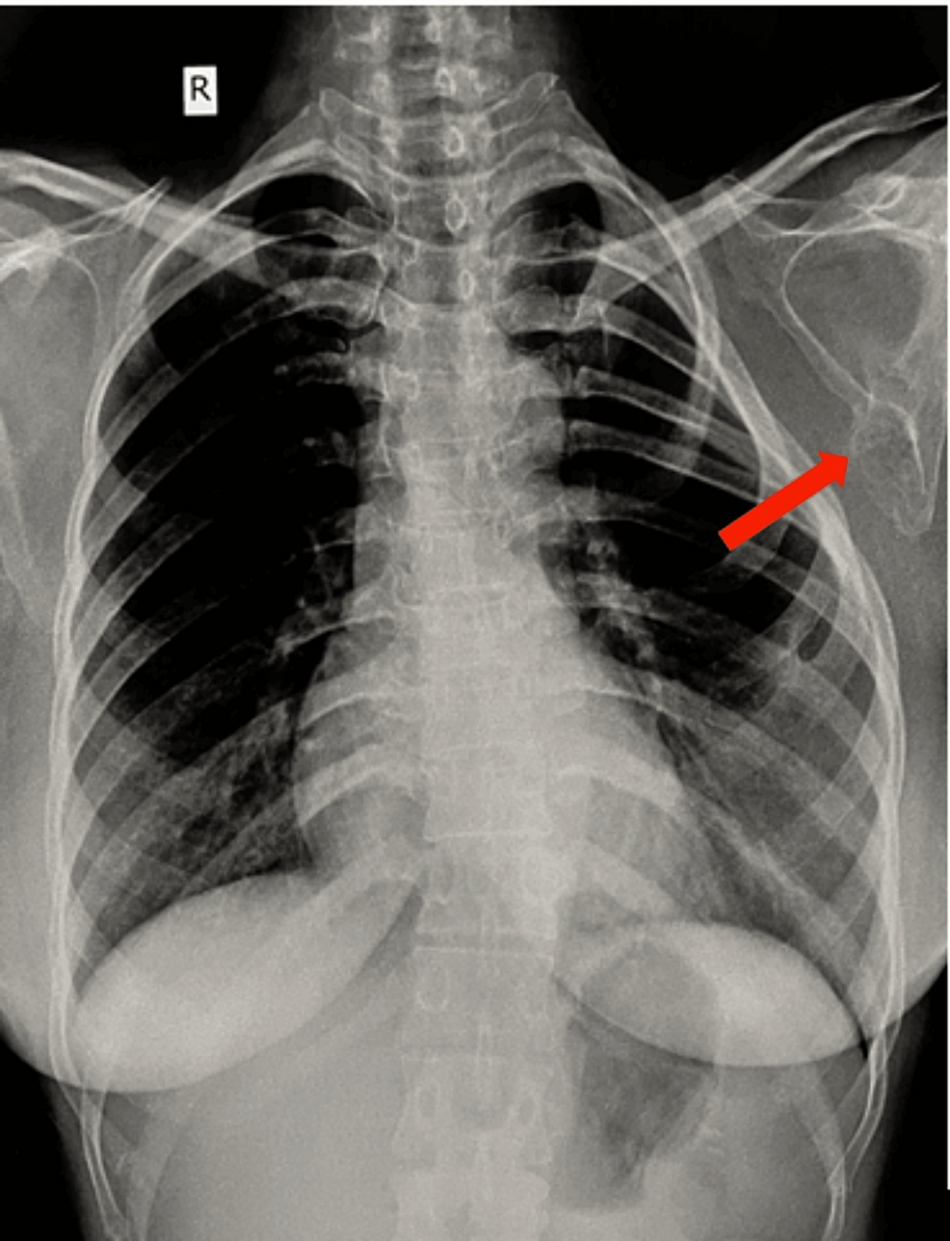
Postoperative X-ray after 6 months showing corrected chest deformity

At the follow-up examination, the patient had no respiratory complaints or pain in the supra mammary region or axilla. The shoulder and scapular movements were in full range without pain. The scapulothoracic movements returned to normal range after four weeks on the first follow-up. The chest wall deformity took six months to get resolved entirely after surgery. There were no respiratory or other symptoms or complaints, and the patient was comfortable. Postoperative plain radiograph after six months showed the corrected chest deformity (Figure 14).

## Discussion

Osteochondromas are the most common benign bone tumor [5,6]; 14% of these tumors are hereditary multiple OC (autosomal dominant disorder), and 86% are solitary OC [6,7]. Osteochondromas constituted 35.8% of benign bone tumors and 8.5% of all bone tumors reported in a retrospective study from Mayo Clinic [8]. It is usually located in the metaphyseal region of the long bones, while the involvement of flat bones is less common [5]. Of note, 90% occur in the distal femur, tibia, or humerus, as these areas are the most actively growing ends of the long bones. The scapula as the site accounts for 1-3% of all primary bone tumors [2]. OC is the most common primary bone tumor of the scapula, with an incidence rate of 4.6%. Most osteochondromas are asymptomatic, and symptoms often depend on the size and location of the lesion [5].

OC is a non-tender, slow-growing, painless cosmetic deformity. Pain is caused by complications such as fracture, bursa formation, arthritis, and impingement of adjacent structures like blood vessels, tendons, nerves, or spinal cord [5,6]. Scapular OC usually occurs on the ventral surface and causes pain and grating sensation with the scapula movements as part of scapulothoracic articulation [9]. Decreased range of motion of the shoulder joint and pseudo-winging of the scapula are expected [10]. Routine radiographs and CT images usually establish the diagnosis, and a CT scan is essential in defining the extent and location of the scapular osteochondromas, which is crucial in planning the surgical excision.

In the present case, an anteroposterior radiograph was obtained, which delineated the chest wall deformity and showed the displaced ribs. CT scan images and 3D CT reconstruction images were helpful in precisely defining the extent of the tumor and chest wall deformity, as well as the compressive effects on the left lung. Chest wall deformity due to scapular osteochondroma was previously reported by Hiroyasu Tomo et al. in 2004. In our case, too, the patient presented with respiratory complaints as the lung was compressed at three-fourths of the capacity, and this was precisely shown through CT scan images and helped in planning the surgical removal. The present case is the second case to report chest wall deformity. The treatment of osteochondroma involves complete surgical excision of the tumor; the cartilage cap should be removed to prevent a recurrence. Any suspicion of malignancy and cosmetic disfiguring can be indications for the surgical removal of the tumor mass.

A solitary osteochondroma has been reported to carry a 1-2% risk of developing into chondrosarcoma. In patients with multiple osteocartilaginous exostosis, the risk increases to 5-25% [11]. In ultrasonographic studies, the significance of the thickening of the cartilaginous cap in osteochondroma to diagnose malignant transformation has been described. The lesion is considered benign when the cap is thinner than 1 cm and equivocal when the cap is between 1 and 2 cm; a malignant transformation is considered when the cap is thicker than 2 cm [12]. In this case, the most significant challenge was removing the tumor mass without damaging the pleura, which was more attached to the inferior and medial border. After the tumor mass was identified, it was made free from the soft tissue attachments on the anterior and lateral aspects; the stalk was identified and exposed so that the tumor mass would be excised at the base of the stalk. After cutting the tumor at the base, it was lifted with a bone hook, which made it possible to detach the attachments over the inferior and medial border. Finally, the tumor was removed intact without damaging the pleura.

The prognosis after surgical removal is generally excellent in these patients. In the majority of cases, patients regained the full range of motion of the shoulder joint and arm without any pain. Our patient's shoulder and arm movements were painless and comfortable postoperatively and in full range. The respiratory distress improved in the postoperative period. The chest wall deformity was remodeled after the compressive effects were gone after the decompression was achieved through the removal of the tumor. Chest wall deformity was remodeled and corrected at the second follow-up after two months. At the six-month follow-up, the patient was comfortable with no respiratory complaints, had a full range of motion at the shoulder joint, and the chest wall deformity had almost been remodeled and corrected.

## Conclusions

While osteochondromas are common bone tumors, scapular osteochondromas arising from the ventral surface are rare entities. The clinical presentation should be examined in detail, and all complaints should be noted, which can provide vital clues for an appropriate and timely diagnosis. Surgical removal should be planned accordingly and surgeons should be prepared to deal with the difficulties likely to be encountered intraoperatively. Postoperative chest physiotherapy and shoulder mobilization are essential to rectify chest deformity and to fix shoulder function so that it returns to the normal range of movements.
